# The Relationship between Body Composition and ECG Ventricular Activity in Young Adults

**DOI:** 10.3390/ijerph191711105

**Published:** 2022-09-05

**Authors:** Elena Ioana Iconaru, Constantin Ciucurel

**Affiliations:** Department of Medical Assistance and Physical Therapy, University of Pitesti, 110040 Pitesti, Romania

**Keywords:** bioimpedance analysis, electrocardiography, systolic and diastolic intervals, young people

## Abstract

This study aimed to determine the correlation between body composition (measured as weight, body mass index, and body fat percentage (BFP)) and electrocardiographic ventricular parameters (the QT and TQ intervals and the ratios between the electrical diastole and electrical systole (TQ/QT) and between the cardiac cycle and electrical diastole (RR/TQ), both for uncorrected and corrected intervals) in a sample of 50 healthy subjects (age interval 19–23 years, mean age 21.27 ± 1.41 years, 33 women and 17 men). Subjects’ measurements were performed with a bioimpedancemetry body composition analyzer and a portable ECG monitor with six leads. Starting from the correlations obtained between the investigated continuous variables, we performed a standard linear regression analysis between the body composition parameters and the ECG ones. Our results revealed that some of our regression models are statistically significant (*p* < 0.001). Thus, a specific part of the variability of the dependent variables (ECG ventricular activity parameters for corrected QT intervals) is explained by the independent variable BFP. Therefore, body composition influences ventricular electrical activity in young adults, which implies a differentiated interpretation of the electrocardiogram in these situations.

## 1. Introduction

### 1.1. The Principle of Bioimpedancemetry for Assessment of Body Composition in Epidemiological Studies

Measuring body composition in the human body is a common method of determining anthropometric parameters that are correlated with the state of health. Starting from the increases or decreases in these variables, by comparison with standardized nomograms, clinical diagnoses can be formulated with implications for elaborating specific therapeutic interventions [1]. Bioelectrical impedance analysis (BIA) is a widespread method for the reliable determination of body composition in laboratory and/or field conditions, with small intra- and interobserver variability [2]. The method is focused on measuring the human body’s resistance to a very small alternating electrical current based on equations to predict the amount of total body water, fat-free mass, and fat mass [3,4]. Thus, for a specific population, through precise formulas, we can calculate the lean and fat mass of the body, starting from the statistical associations based on biological relationships [3]. Numerous studies have shown that the values obtained through BIA reflect good concordance between a high body mass index (BMI) and high fat [5].

BMI is the most used parameter for defining the anthropometric height/weight characteristics of the population and for classifying individuals into different groups [6]. At the same time, BMI represents a gross and indirect parameter of body fat [7] but is not sufficient for an accurate measure of fatness [5]. BIA has imposed itself as a complementary epidemiological technique, with increased clinical utility, to assess body composition [8].

Recently, BIA field assessment methods have been optimized by the use of lightweight handheld portable devices. These instruments allow quick, accurate, noninvasive, and easy measurement of BFP. Some authors have also shown excellent agreement between hand-to-hand BIA assessment and dual-energy X-ray absorptiometry (DXA)-derived measurements across groups of individuals [9]. For this reason, the model is accurate for the estimation of body composition in epidemiological studies for a specific population because it seems to accurately estimate BFP in controlled conditions for healthy and euvolemic adults [10].

The variables extracted from the BIA and especially the BFP have been associated in various ways with specific indicators of health status and contextual variables. Thus, significant correlations have been established in epidemiological studies between BF and traditional cardiovascular risk factors and cardiovascular diseases [11,12], obesity [13], bone mineralization, and the risk of osteoporosis [14], general mortality [15], health-related quality of life and depression [16], physical activity [17], metabolic health [18], dietary habits [19], growth and development, nutrition, socioeconomic level [20], etc.

One of our distinct interests was to assess the relationship between body composition parameters and cardiovascular homeostasis. Changes in body composition such as the accumulation of adipose tissue and the onset of obesity have potentially harmful effects on the cardiovascular system. Moreover, BFP is an excellent predictor of maximal oxygen consumption (VO2max) [21] and cardiovascular risk, better than BMI [22]. Consequently, BIA is useful for the clinical prediction and diagnosis of cardiovascular diseases. For example, BFP is significantly lower in individuals with normal systolic function than in those with defective arterial compliance [23].

However, things seem to be different in the case of older people or individuals with chronic diseases. In this situation, the so-called paradox of obesity has been highlighted. According to this paradox, obesity for the respective patients may be protective and associated with decreased mortality [24]. Moreover, the increase in BFP predicts a lower risk of short-term cardiac events in patients diagnosed with different types of heart failure [25]. Therefore, the relationship between BFP and cardiovascular function must be analyzed for specific population groups, and generalizations must be carefully argued, taking into account certain categorical variables.

Another field of investigation with multiple practical applications refers to the study of the impact of changes in body composition on electrical heart activity. A common finding of some research is that obesity and an increase in BFP are associated with a higher QT interval of the electrocardiogram (ECG) in young males [26]. Even in healthy normal-weight subjects, increased BMI is related to some electrocardiographic alterations: increasing P-wave length, leftward shifting of the electrical axis of the heart, and decreasing Sokolow–Lyon index [27].

From another perspective, an increase in adipose tissue in the human body interferes with ECG results because the fat mass exerts a high electrical resistance and reduces the voltage of the ECG waves, especially for ventricular activity [28]. Starting from these findings, a model for predicting BFP through sex-based ECG signals based on artificial intelligence has recently been proposed [29]. All these aspects open new perspectives for investigating the complex relationship between body composition and electrical activity of the heart to refine the ECG diagnosis in case of changes in body fat mass in various physiological or pathological situations.

### 1.2. The Utility of Portable ECG Monitoring Devices for Ventricular Activity Recording

Recently many accessible, low-cost technological solutions for healthcare monitoring have been developed, such as mobile-based devices for ECG recording, with a smartphone, tablet, or smart-watch interface [30]. With their help, daily ECG recordings can be made, in different settings, with minimal instruction. Thus, the collected data can be processed and visualized in real time by accessible software healthcare applications, archived, and eventually transmitted to specialized medical staff [31]. Usually, with the help of portable and handheld single-lead ECG devices, heart rate and heart rate variability can be calculated [32]. These portable systems for ECG monitoring are useful for the diagnosis and management of arrhythmias [33]. For this purpose, the manufacturers are striving for good data reliability and optimizing signal quality [31].

Several companies have proposed portable ECG devices with a smartphone interface that allow simultaneous recording on six leads. Preliminary data indicate that the measured electrical intervals are close enough to allow the detection of clinically meaningful abnormalities [34]. These devices have good accuracy for reliable QT and QTc interval determination and, therefore, can be used in clinical trials [35]. In addition, this ECG recording variant is useful for obtaining information on cardiac rhythm and electrical intervals for patients who have difficulty achieving the classic 12-lead ECG, such as the existence of mental health diseases [36]. In contrast, these methods are less reliable for determining left ventricular hypertrophy, QRS wave amplitudes, or ischemic changes [37].

In standard or fast ECG recordings based on portable devices, data on ventricular activity are of great interest for clinical diagnosis. Conventionally, ventricular electrical activity refers to the systolic and diastolic parameters. The QRS complex is the most prominent feature of an ECG signal [38] and represents the deflection determined by ventricular depolarization [39]. The automatic ECG analysis algorithm for clinical diagnosis is usually based on QRS detection [40]. On the ECG, systolic and diastolic ventricular activity can be overlapped with the durations of the QT and TQ intervals, respectively, because there is a correspondence between the electrical and mechanical phenomena of the heart [41]. Moreover, the ratio of QT to TQ interval within a cardiac cycle (QT/TQ ratio) is a surrogate of systolic–diastolic interval interaction and reflects the balance of cardiac contraction and relaxation for one heartbeat [42]. In the same sense, the ratio between the cardiac cycle and electrical diastole (RR/TQ) is another element that has been correlated with the harmonic rhythm of the physiological activity of the heart [43].

In conclusion, most clinical research is performed using conventional 12-lead ECG devices, which raises technical and time-consuming issues for recordings. As an alternative, handheld portable ECG technology can accurately screen a few cardiac abnormalities in the absence of conventional ECG devices in various healthcare settings [44]. Regarding research into the correlations between body composition and ECG data, we did not identify any study reporting the use of portable ECG devices with smartphone interfaces as screening methods for the measurements performed. Thus, research into systolic and diastolic electrical intervals and the ratio between them using a portable ECG device, considering body composition variables, brings novelty elements for clinical diagnosis.

## 2. Materials and Methods

### 2.1. Aim of the Study and Premise

The present research aimed to investigate the body composition and electrical activity of the heart in a representative sample of young adults. We selected participants without significant pathological history, but with variable body weight, including cases of overweight and obesity. Through this study, we wanted to determine the association between the variables determined by the BIA method (weight (W), BMI, and BFP) and the electrical ventricular activity of the heart (the QT and TQ intervals and the ratios between the electrical diastole and electrical systole (TQ/QT) and between the cardiac cycle and electrical diastole (RR/TQ), both for uncorrected and corrected intervals), evaluated using a portable ECG recorder with six leads.

### 2.2. Participants and Type of Study

The experimental design of the research was an observational cross-sectional study on a sample of young adults (*n* = 50, mean age 21.27 ± 1.41 years, 33 women and 17 men). All subjects gave written informed consent to participate in the study, according to the ethical principle of research with humans. The research was approved by the ethics committee of the Research Center for Promoting Excellence in Professional Training, University of Pitesti (reference number 142/7 March 2022).

To include the participants in the study, we applied the following selection criteria: age between 18 and 23 years, clinically healthy after a medical examination, variable body weight (including obese individuals, but without other metabolic complications), and without significant pathological antecedents that may affect the nutritional status [45]. All subjects were normotensive and without a history of hypertension. In addition, for female subjects, the exclusion criteria were a pregnancy or breastfeeding state [46]. The subjects were students from the University of Pitesti. We aimed to have this narrow age range of the participants because we wanted to exclude the influence of the age factor on body composition and electrocardiographic parameters [47,48].

### 2.3. Data Acquisition

The study required successive evaluations according to standard procedures for each participant to determine body composition parameters and record ECG data. All measurements were carried out between 8 and 10 a.m. by the same researcher trained in the operation of the devices. The participants were tested on an empty stomach. In addition, subjects were recommended to have at least 7 h of sleep the previous night, a normal diet in the last 48 h without excesses, and a normal physical activity regime during the last 3 days. All participants were free from physical trauma or any physiological and psychological condition that would interfere with the testing procedure, including the recent administration of diuretics or any medication [49]. We opted for BIA determination of body composition before breakfast, as eating leads to a slight decrease in bioelectrical impedance and, consecutively, in BFP [50].

Subjects were initially weighed using a portable digital scale (Omron HN-286, Omron Corporation, Kyoto, Japan) to the nearest 0.1 kg. Then, body height (H) was determined with a portable stadiometer (Seca 213, Seca GmbH & Co., Kg, Hamburg, Germany) to the nearest 0.001 m.

Body composition was determined using a handheld Omron body fat monitor, model HBF 306, Omron Healthcare Co., Ltd. Dalian, China. The device is accurate for field use. The obtained BFP values are similar to those provided by the dual-energy X-ray absorptiometry (DEXA) method [51], and the method has very good test–retest reliability [52]. BFP was measured with the device placed in the hands based on electric resistance and personal categorical data (age, sex, W, and H). According to the manual of instructions, the participant must stand in the orthostatic position, with both feet slightly apart and with both arms extended straight out at a 90-degree angle to the body, parallel to the floor, with both hands placed on the device by holding the sensors. During the assessment, no movements of the body were allowed. The monitor displays the following parameters: BMI (calculated as weight (kg)/height^2^ (m^2^)) and BFP. Two measurements were performed in approximately 2 min for each subject, and the mean BFP was calculated [53].

The ECG was recorded for each participant after the protocol of body composition assessment. Each subject was placed at rest in the clinostatic position for 5 min, and then an Istel HR-2000 ECG Recorder (Diagnosis S.A., Bialystoc, Poland) was applied to the body at the height of the sternum. This portable device is a six-lead ECG recorder (leads I, II, III, aVR, aVL, aVF) with four built-in electrodes, compatible with iOS and Android operating systems. The ECG records are sent to mobile devices via Bluetooth through a software application (Istel ECG App, version 3.12, Diagnosis S.A., Bialystoc, Poland). The device generates a report that is exported as an attachment in pdf format. For the measurement process, we selected a length of 30 s [54], high quality of the test (the recommended frequency by the manufacturer of 320 Hz), and a normal sensitivity of the electrodes. The Istel HR-2000 ECG has good accuracy for detecting atrial fibrillation during daily 30 s ECG recordings [55] and might serve for the identification of other types of arrhythmias (supraventricular, ventricular, or atrioventricular blocks) [56]. The Istel HR-2000 ECG is suitable for arrhythmia diagnosis and QT and RR interval measurements [57].

We manually measured the RR and QT intervals from the recorded ECGs. Then, we calculated the heart rate (HR) and the corrected QT intervals (QTc), the TQ and TQ corrected intervals (TQc), and the ratios between the electrical diastole and electrical systole (TQ/QT and TQc/QTc) and between the cardiac cycle and electrical diastole (RR/TQ and RRc/TQc). For RR determination, we measured the duration between two consecutive R waves. The measurement of the QT intervals was performed using the tangent method, which has good reproducibility and inter-rater reliability. The steepest slope of the final descending limb of the T wave is identified. From this point, a tangent is drawn. The QT interval represents the distance from the beginning of the QRS complex to the point where the tangent crosses the isoelectric line [58].

Using the Bazett formula, we calculated the QTc value [59]:

QTc = QT/RR^1/2^

If we consider QT and QTc as intervals that reflect the ventricular electrical systole, then we can use the TQ and TQc intervals as the equivalent of ventricular electrical diastole, according to the following formulas:

TQ = RR − QT

TQc = RRc − QTc = 1000 − QTc

In the last formula, RRc represents the standard value of 1000 ms. The ECG interval measurements were conducted in lead II. We calculated the ECG parameters during stable sinus rhythm on three successive heartbeats, and we considered the average values of the data [59].

### 2.4. Outcomes and Statistical Analysis

The data were statistically processed by descriptive and inferential statistics using IBM SPSS 20.0 software (IBM Corp., Armonk, NY, USA) [60]. Firstly, we determined the mean, the standard deviation (SD), the minimum and maximum values, and the coefficient of variation (CV) of the data. Next, we ran a correlational analysis of data (determination of parametric Pearson’s correlations) to highlight the relationship between the variables of body composition (W, BMI, and BFP) and the electrocardiographic ventricular variables (HR, QT, QTc, TQ, TQc, RR, TQ/QT, RR/TQ, TQc/QTc, and RRc/TQc). Finally, after checking the required assumptions, we conducted a simple linear regression analysis for the variables with relevant associative relationships. The SPSS regression analysis included the ANOVA test, which describes how well the regression models fit the data. We applied the Durbin–Watson test to check the independence of observations. For the assumptions of the normal distribution of residuals (errors), we used the histograms method (with a superimposed normal curve) and the normal probability plot (P-P) method [61]. The histogram method allows to visually check if the residuals are normally distributed and follow a bell-shaped curve. A normal P-P plot is based on a graphical comparison of a data set with a normal distribution.

## 3. Results

The summary results of body composition analysis and ECG records in the group of participants are presented in Table 1 and Table 2.

Overall, our results revealed that the average nutritional status of the subjects belonged to the normal weight category (mean BMI = 24.38 ± 5.11), according to the NIH/WHO guidelines for BMI [62]. Thus, 4% of the subjects were underweight, 64% had normal weight, 18% had preobesity, 10% had obesity class I, and 4% had obesity class II. In addition, according to age and sex nomograms for BFP [63,64], 4% of the subjects had low body fat, 46% had normal body fat, 22% had high body fat, and 28% had very high body fat.

Regarding the ECG records, all subjects were in sinus rhythm, without pathological changes, the average heart rate in the group being 77.76 ± 14.03 beats/min. Classically, normal QTc in a standard 12-lead ECG is generally considered to be between 350 and 440 ms, but the QTc is influenced by sex [65]. For our participants, the determined QTc intervals were within normal limits for 98% of the subjects and above the normal limits for 2% of them, according to the sex reference values: QTc < 440 ms for adult males and <460 ms for adult females [66].

Next, we determined Pearson’s correlation coefficient R and its statistical significance to quantify the strength and direction of the linear relationship between pairs of numerical variables (Table 3). Thus, the most important correlations for our study were between BFP and QTc (R = 0.55), TQc (R = −0.55), TQc/QTc (R = −0.55), and RRc/TQc (R = 0.55). These values correspond to moderate correlations in intensity according to the rule of thumb [67]. Interestingly, the correlations between BMI and the ECG variables mentioned above are lower, although both BMI and BFP are parameters used in clinical practice to predict adiposity [68]. We excluded from this analysis the very high correlations between parameters that resulted from the same calculation formulas.

After the correlation analysis, linear regression was the next step for the statistical processing of data for the significant correlations. Thus, we ran a standard linear regression analysis between the BFP and the ECG variables QTc, TQc, TQc/QTc, and RRc/TQc (Table 4). The necessary assumptions that were met were: the variables were continuous, there was a linear relationship between the pairs of variables, there were no significant outliers, we had the independence of observations, and the residuals (errors) of the regression line were approximately normally distributed.

The obtained regression coefficients were statistically significant for all models and reflected the unstandardized effect size in terms of the strength of the relationship between the independent and the dependent variables [69]. The following regression equations summarize our results:

QTc = 339.5 + BFP × 1.79

TQc/QTc = 1.89 − BFP × 0.11

RRc/TQc = 1.5 + BFP × 0.005

In conclusion, the regression models for BFP (independent variable) significantly predicted each dependent variable: QTc, TQc, TQc/QTc, and RRc/TQc. The R square values from Table 4 show how much of the total variation in the dependent variables was attributed to BFP.

## 4. Discussion

Following this research, we confirmed the hypothesis according to which there is a correlation of moderate intensity between BFP, as a specific parameter of body composition, and the ECG electrical intervals corresponding to ventricular systole and diastole, respectively the ratios TQc/QTc and RRc/TQc. In contrast, the association between BMI, which is the classic marker of metabolic/nutritional status, with the same ECG indicators was lower. Moreover, the results of the regression analysis revealed statistically significant relationships between the investigated variables. Therefore, we were able to provide an estimate of the magnitude of the impact of a change in BFP on ventricular electrical parameters.

ECG is influenced by morphological changes induced by obesity, but conduction intervals (duration of the P wave, QRS complex, and the PQ interval) are not affected by weight loss [70]. It is well known that the adipose tissue of the human body, especially from the thoracic region, lowers the electric potentials from unipolar leads because it acts as an electric insulation layer [71]. Beyond the possible interference of adipose tissue with the technical process of capturing the electrical potentials of the heart, changes in electrical intervals are present from the stage of a healthy young adult. Thus, we can conclude that healthy young adults with increased BFP have electrical ECG changes, such as those mentioned, in a clinically unapparent form but detectable by ECG.

Among the ECG changes that may occur in obese patients is the lengthening of the QTc interval [70], with a linear relationship between QTc and BFP. Although the QTc may not be extremely increased (≈440 ms) in these people, assessment of QTc lengthening may be predictive of increased mortality rates in a healthy obese population [70].

In addition, these results come from a fast field experimental design, which involved the use of portable devices for screening participants. The BIA method is widely used in research that uses body fat monitors to quickly determine body composition. Instead, the portable ECG with six leads, with smartphone applications, has recently appeared on the market and has been less used for studies on the electrical intervals of the heart [34]. Moreover, the use of both types of assessment for the assumed objectives of the research is a novelty. Our results are encouraging and justify the extended discussions.

An important aspect to discuss is the causes of the association of QT prolongation with BFP increase. Several studies have shown that QT interval prolongation is common in obesity, and it shortens with weight loss [26,71]. It seems that the distribution of adipose tissue also influences the values of the QT interval, with upper body obesity exerting a more marked effect [72]. Moreover, in the case of severe obesity, QT prolongation is associated with lower serum calcium levels as a consequence of impaired calcium/phosphate metabolism [73]. The treatment itself for obesity may aggravate or prolong QTc due to the induced electrolyte imbalances [74].

Classically, studies on ECG changes induced by obesity or adipose tissue growth refer to people with a specific metabolic—nutritional pathology. Given that the subjects investigated in our study were healthy, we can assume that these ECG changes appear, even at a young age, in healthy people but without a normal weight status. The same issue remains under discussion: to what extent a person with a particular subphenotype, who does not have normal values of body weight, can be considered healthy or shows latent pathological elements, which are visceral metabolic complications [75]? In other words, the proven cardiovascular risks in obese people could be present in a subclinical format in healthy young people with high BFP values. Our results are consistent with those of authors who reported that the health status of people with increased adiposity is uncertain [76].

Our results suggest several potential directions for further study. The monitoring of subjects with simultaneous increases in BFP and QTc interval, which hallmarks ventricular repolarization impairment and is a risk factor for the development of acute cardiac events, becomes essential [74]. To this end, the use of fast methods of screening body composition and the electrical activity of the heart, with the help of portable devices, becomes attractive for field investigations. In addition, starting from the statistically significant model of prediction of the ECG ventricular indicators based on body composition status, BFP obtained through routine BIA measurement can identify adults at increased risk for QTc prolongation.

Another contribution of our study, with a novelty note, concerns the association between BFP and the ratios between the electrical diastole and electrical systole (TQc/QTc) and between the cardiac cycle and electrical diastole (RRc/TQc). These ratios represent the synchronized contraction and relaxation operation of the heart, respectively, the noninvasive measure of diastolic function [77]. Our results indicate that for TQc/QTc, an increase in BFP is associated with a decrease in the ratio. Instead, for RRc/TQc, an increase in BFP is associated with an increase in the ratio. The effect of BFP growth on the two reports is similar to acute psychological stress on systolic and diastolic functions [77]. Thus, increased fat mass determines similar effects to acute stress on ventricular electrical activity.

Finally, another important conclusion is the higher association between BFP with the analyzed ECG indicators than between BMI and the same parameters. Therefore, we can say that BFP is a more accurate marker of the risk of impaired ventricular electrical activity of the heart than BMI. Consistent with our results, we can mention another study on the relationship between body fat mass and the left ventricular longitudinal myocardial systolic function in diabetic patients. Thus, the authors of the mentioned study demonstrated that body fat index is more closely associated than BMI with left ventricular longitudinal function [78].

Limitations and strengths of the study. As a limitation, this study lacks generalizability for subgroups of the population depending on sex. In the experimental group, we had more female subjects than male subjects, and the size of the group did not allow statistical analysis by subgroups due to the high risk of inflating the overall type I statistical error rate [79]. Another limitation of the study was that subjects were not specifically screened for diabetes/prediabetes, electrolyte imbalances, or blood lipid profiles. Finally, in this research, we used the ECG as a widely available and cheap method for clinical investigation in obese individuals. It should be noted that for such cases, the method has low sensitivity and specificity [80]. The study also had some notable strengths. One of these was the assessment, through a novel and simple screening design, of body composition and the electrical activity of the heart. Our results, in accordance with the opinions of most researchers, add further arguments for the existence of the association between BFP and electrical ventricular activity in healthy young adults. Another strength was the new, proven relationship between BFP and ECG interval ratios. The possibility of identifying the population at risk for ventricular impairments through a portable BIA device is also a remarkable strength of this research.

## 5. Conclusions

Our experimental design, based on the use of fast methods of screening body composition and the electrical activity of the heart with the help of portable devices, allowed pertinent data to be collected. The obtained results revealed that the regression models for BFP (independent variable) and the dependent variables QTc, TQc, TQc/QTc, and RRc/TQc were statistically significant (*p* < 0.001). Therefore, body composition influences ventricular electrical activity in young adults, which implies a differentiated interpretation of the electrocardiogram in these situations and the risk for ventricular impairments.

## Figures and Tables

**Table 1:** Statistical indicators for body composition parameters in the experimental group (*n* = 50).

Variable	Age (Years)	H (cm)	W (kg)	BMI (kg/m^2^)	BFP (%)
Mean	21.27	168.50	69.53	24.38	28.03
SD	1.41	8.70	16.34	5.11	8.83
CV (%)	6.63	5.17	23.51	20.97	31.51
Min	19	152.3	45.7	16.07	9.4
Max	23	194.7	110.3	39.45	49.8

Note: W, weight; H, height; BMI, body mass index; BFP, body fat percentage; SD, standard deviation; Min, minimum value; Max, maximum value; CV, coefficient of variation; *n*, group size.

**Table 2:** Statistical indicators for ventricular electrical parameters in the experimental group (*n* = 50).

Variable	HR (Beats/min)	QT (ms)	TQ (ms)	QTc (ms)	TQc (ms)	RR (ms)	TQ/QT	RR/TQ	TQc/QTc	RRc/TQc
Mean	77.76	345.32	449.98	389.69	610.31	795.30	1.30	1.82	1.58	1.64
SD	14.03	29.80	119.41	28.93	28.93	136.77	0.30	0.23	0.18	0.08
CV (%)	18.04	8.63	26.54	7.42	4.74	17.20	23.29	12.68	11.57	5.02
Min	58	280	208	346	504	508	0.61	1.55	1.02	1.53
Max	118	400	657	496	654	1034	1.82	2.63	1.89	1.98

Note: HR, heart rate; QT, TQ, RR, ECG electrical intervals; QTc, TQc, RRc, ECG-corrected ECG electrical intervals; SD, standard deviation; Min, minimum value; Max, maximum value; CV, coefficient of variation, *n*, group size.

**Table 3:** Correlation output (Pearson’s correlation coefficient R) between the recorded variables and level of statistical significance *p* (*n* = 50).

Variable	W	H	BMI	BFP	HR	QT	TQ	QTc	TQc	RR	TQ/QT	RR/TQ	TQc/QTc	RRc/TQc
**W**	1.00													
**H**	0.50 *	1.00												
**BMI**	0.88 *	0.03	1.00											
**BFP**	0.30 *	−0.49 *	0.61 *	1.00										
**HR**	0.18	0.05	0.16	0.14	1.00									
**QT**	−0.06	−0.26	0.10	0.33 *	−0.65 *	1.00								
**TQ**	−0.14	0.05	−0.17	−0.23	−0.96 *	0.50 *	1.00							
**QTc**	0.11	−0.27	0.28 *	0.55 *	0.42 *	0.41 *	−0.58 *	1.00						
**TQc**	−0.11	0.27	−0.28 *	−0.55 *	−0.42 *	−0.41 *	0.58 *	−1.00 *	1.00					
**RR**	−0.13	−0.02	−0.12	−0.13	−0.98 *	0.65 *	0.98 *	−0.42 *	0.42 *	1.00				
**TQ/QT**	−0.15	0.13	−0.23	−0.37 *	−0.87 *	0.22	0.95 *	−0.80 *	0.80 *	0.88 *	1.00			
**RR/TQ**	0.20	−0.11	0.28	0.40 *	0.84 *	−0.15	−0.89 *	0.82 *	−0.82 *	−0.81 *	−0.95 *	1.00		
**TQc/QTc**	−0.10	0.27	−0.27	−0.54 *	−0.42 *	−0.41	0.58 *	−0.99 *	0.99 *	0.41 *	0.80 *	−0.80 *	1.00	
**RRc/TQc**	0.12	−0.27	0.29 *	0.54 *	0.42	0.40	−0.58 *	1.00 *	−1.00 *	−0.42 *	−0.78 *	0.83 *	−0.98 *	1.00

Note: W, weight; H, height; BMI, body mass index; BFP, body fat percentage; HR, heart rate; QT, TQ, RR, ECG electrical intervals; QTc, TQc, RRc, ECG-corrected electrical intervals; * *p* < 0.05 was considered statistically significant (2-tailed); *n*, group size.

**Table 4:** Model summary, ANOVA report, and coefficients for simple linear regression analysis—BFP versus electrical ventricular parameters (*n* = 50).

Variable	R	R Square	Adjusted R Square	SE	F	*p*	β0	SE	*p*	95% LB	95% UB	β1	SE	*p*	95% LB	95% UB
**QTc**	0.55	0.3	0.29	24.48	20.52	0.001	339.5	11.63	0.001	316.12	362.88	1.79	0.4	0.001	1	2.6
**TQc**	0.55	0.3	0.29	24.48	20.52	0.001	660.5	11.63	0.001	637.12	683.88	−1.79	0.4	0.001	−2.59	−1
**TQc/QTc**	0.54	0.29	0.28	0.15	19.88	0.001	1.89	0.07	0.001	1.75	2.04	−0.11	0.003	0.001	−0.16	−0.006
**RRc/TQc**	0.54	0.29	0.28	0.07	20.03	0.001	1.5	0.03	0.001	1.43	1.57	0.005	0.001	0.001	0.003	0.007

Note: QTc, TQc, RRc, ECG-corrected electrical intervals; R, Pearson’s coefficient of correlation; SE, standard error; F, test for overall significance for the linear model; *p*, level of statistical significance; β0, the intercept coefficient; β1, the regression coefficient; 95% LB and 95% UB, lower bound and upper bound of the 95% confidence interval; *n*, group size.

## Data Availability

The data are available on request from the corresponding author. All data relevant to the study are included in the article.
